# Right-sided colon endoscopic hand suturing with a single intubation using readily available endoscopic equipment

**DOI:** 10.1055/a-2674-4623

**Published:** 2025-08-27

**Authors:** Ka Luen Thomas Lui, Wai Pan Elvis To

**Affiliations:** 1 667036 Department of Medicine, School of Clinical Medicine, Li Ka Shing Faculty of Medicine, The University of Hong Kong, Hong Kong, Hong Kong

This case report details a simplified and potentially safer method for endoscopic hand suturing in the right-sided colon, eliminating the need for cumbersome equipment changes during the procedure ( Video 1 ).

Right-sided colon suturing technique performed endoscopically during a single intubation, using readily available equipment.Video 1

Endoscopic hand suturing in the right colon presents a significant challenge in safely managing the sharp needle. While previous methods have utilized an oblique distal attachment to shield the needle tip, this approach necessitates multiple colonoscope insertions and withdrawals to attach and detach the device for suturing 1 . This process leaves the sharp needle unattended within the colon, creating a risk of unnoticed injury.

This new case report describes a successful alternative. A 60-year-old male underwent endoscopic submucosal dissection (ESD) for a 3-cm lateral spreading tumor granular type (LST-G) in the ascending colon ( Fig. 1 and Fig. 2 ). The resulting post-ESD defect was closed using a 17-mm V-loc 180 absorbable barbed suture (Covidien, Mansfield, Massachusetts, USA) delivered with a flexible needle holder (SutuArt; Olympus, Tokyo, Japan) through a standard colonoscope fitted with a straight, soft distal attachment (Olympus, Tokyo, Japan) ( Fig. 3 , Fig. 4 , Fig. 5 ).

**Fig. 1:**
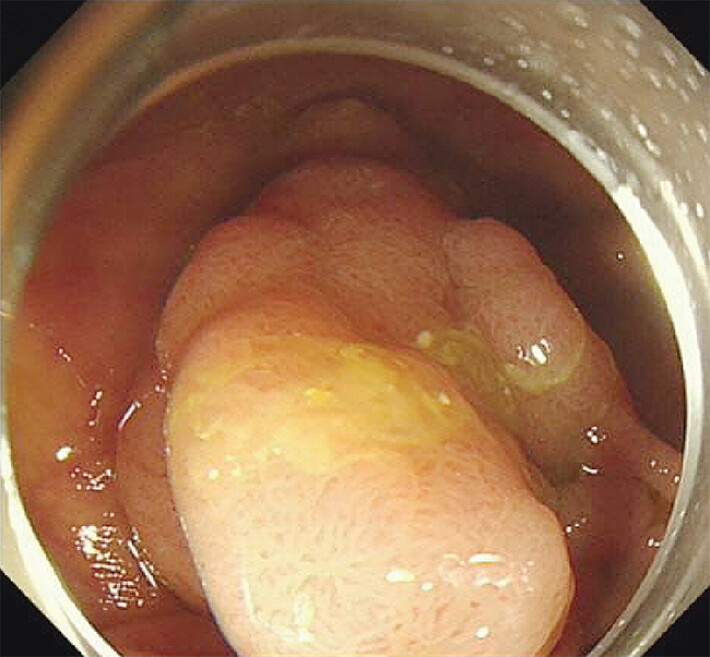
A 3-cm LST-G at the ascending colon.

**Fig. 2:**
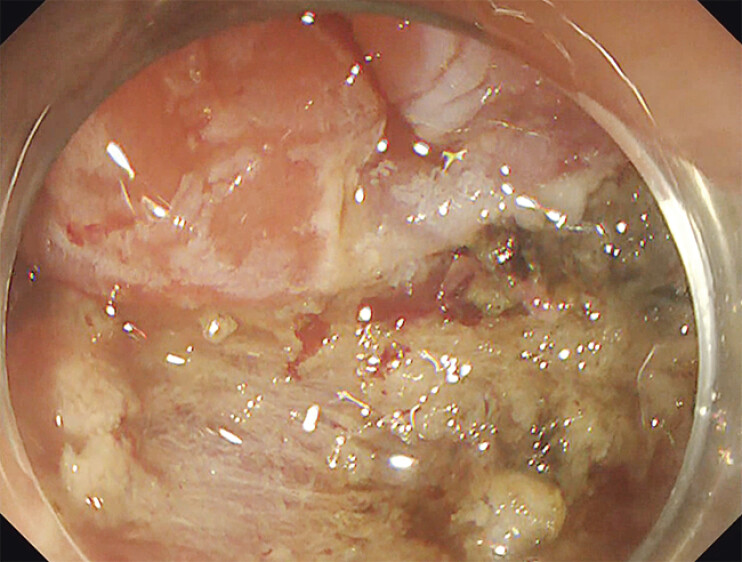
The whole lesion was removed with ESD en bloc, leaving the mucosal defect.

**Fig. 3:**
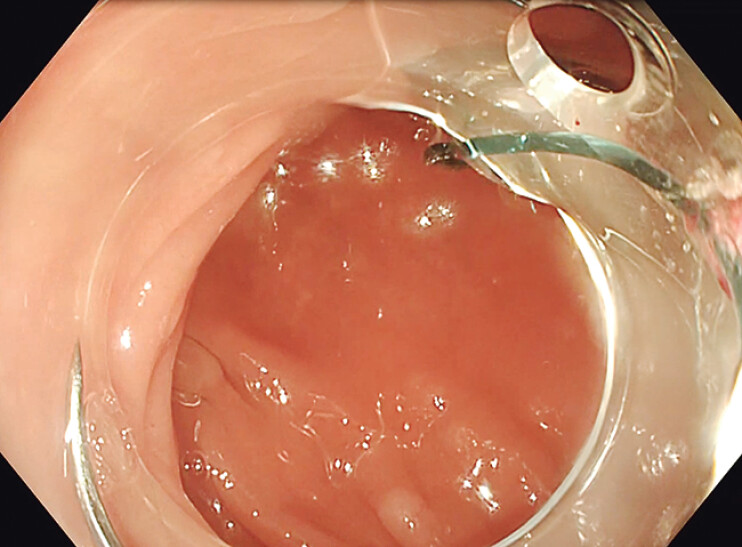
A 17-mm V-loc 180 absorbable barbed suture delivered with a flexible needle holder through a standard colonoscope fitted with a straight, soft distal attachment.

**Fig. 4:**
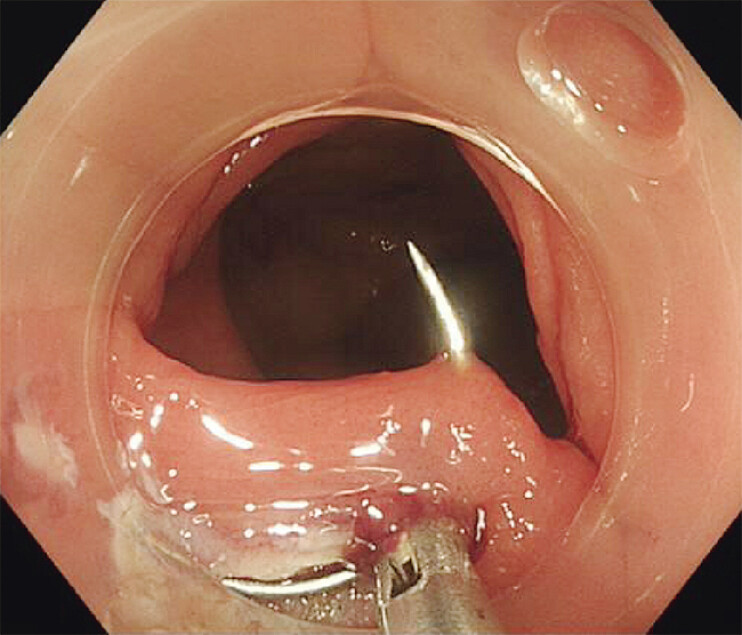
The post-ESD wound was closed with endoscopic hand suture.

**Fig. 5:**
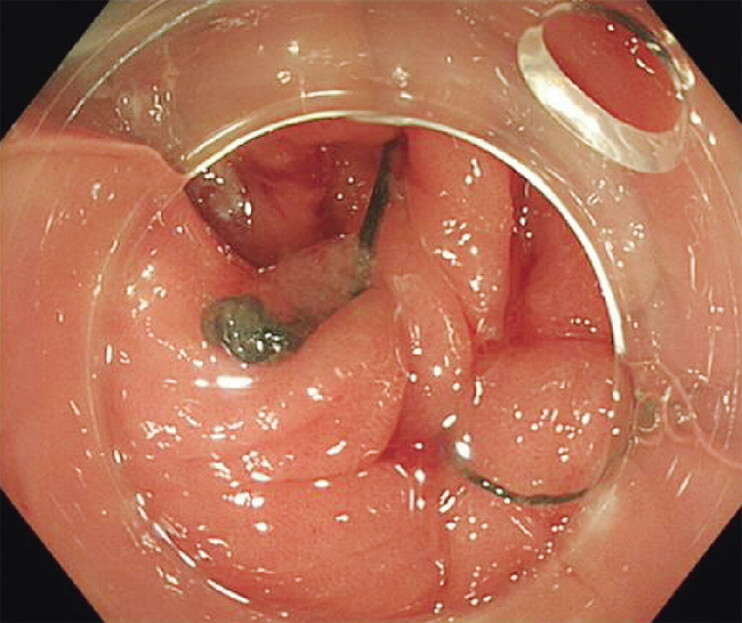
The post-ESD wound was completely closed.

The procedure was completed successfully in a single intubation, and the patient was discharged without complications. The final histology was tubulovillous adenoma with high-grade dysplasia with a clear margin. This case demonstrates the feasibility of performing endoscopic hand sutures in the right colon using readily available endoscopic equipment, potentially streamlining the process and enhancing patient safety by avoiding the risks associated with multiple insertions and an unsecured needle.

Endoscopy_UCTN_Code_CPL_1AJ_2AJ

## Notes

Endoscopy E-Videos https://eref.thieme.de/e-videos
*E-Videos* is an open access online section of the journal *Endoscopy* , reporting on interesting cases and new techniques in gastroenterological endoscopy. All papers include a high-quality video and are published with a Creative Commons CC-BY license. Endoscopy E-Videos qualify for HINARI discounts and waivers and eligibility is automatically checked during the submission process. We grant 100% waivers to articles whose corresponding authors are based in Group A countries and 50% waivers to those who are based in Group B countries as classified by Research4Life (see: https://www.research4life.org/access/eligibility/ ). This section has its own submission website at https://mc.manuscriptcentral.com/e-videos .
